# Genomic and phenotypic analysis of ST25 *A. baumannii* identifies virulence-associated clades and capsular/outer core locus types

**DOI:** 10.1128/msphere.00717-25

**Published:** 2025-12-31

**Authors:** Antonella Migliaccio, Thibault Destanque, Marisa Haenni, Jean-Yves Madec, Keith A. Jolley, Maria Stabile, Eliana De Gregorio, Agnese Lupo, Raffaele Zarrilli

**Affiliations:** 1 Department of Public Health, University of Naples Federico II 165485, Naples, Italy; 2 ANSES - University of Lyon, Unité Antibiorésistance et Virulence Bactériennes 133614 , Lyon, France; 3 Department of Biology, University of Oxford 98459, Oxford, United Kingdom; 4 Department of Molecular Medicine and Medical Biotechnology, University of Naples Federico II 208969, Naples, Italy; University of Nebraska Medical Center College of Medicine, Omaha, Nebraska, USA

**Keywords:** *Acinetobacter baumannii*, ST25 clonal lineage, KL/OCL typing, virulence assays, biofilm formation, resistance to desiccation, resistance to oxidative stress, resistance to serum

## Abstract

The increase in the infection caused by *Acinetobacter baumannii* is sustained by the selection of distinct epidemic clonal lineages, which are frequently resistant to a broad range of antimicrobials and possess virulence traits responsible for their persistence in the contaminated environment and spread among patients. The present study aimed to perform an integrated genomic and phenotypic analysis to assess the virulence features of ST25 isolates. *A. baumannii* isolates assigned to the ST25 epidemic clonal lineage shared high genomic similarity and clustered in four clades (I, II, III, and IV), with clade IV further subdivided into CIVa, CIVb, CIVc, and CIVd. Capsular locus (KL) KL14 was the predominant KL type (47%). Accessory genome analysis showed the presence of tartrate metabolism genes only in CII genomes. CIVb and CIVd ST25 *A. baumannii* isolates showed higher ability to infect *Galleria mellonella* larvae than CI, CII, CIII, and CIVc isolates. Hydrogen peroxide resistance was higher in CI, CII, CIVb, and CIVd isolates compared with CIII and CIVc isolates. In desiccation survival tests, CIII, CIVb, and CIVd isolates exhibited prolonged survival. In addition, CI, CII, CIII, CIVb, and CIVd isolates showed higher serum resistance than CIVc isolates. Also, KL14 type and lipooligosaccharide outer core locus (OCL) OCL6 type isolates were significantly more resistant to oxidative stress, to desiccation, and possessed a high ability to kill *G. mellonella* larvae. A positive and significant correlation was found between AdeB and AdeJ efflux pump expression and hydrogen peroxide resistance.

**IMPORTANCE:**

In this study, we characterized the genotypic and phenotypic features of *A. baumannii* strains assigned to the ST25 epidemic clonal lineage, which were isolated from humans, animals, and the environment. We found that ST25 *A. baumannii* isolates, irrespective of their antimicrobial resistance, showed peculiar virulence features among clades, isolates assigned to clade IVb and IVd showing the highest virulence and elevated resistance to serum and desiccation. Also, a positive significant correlation was found between the presence of KL14 and outer core locus 6 genotypes and resistance to oxidative stress, resistance to desiccation, and the ability to kill *G. mellonella* larvae. Phenotypic differences reflected clade identity rather than isolate origin, suggesting that specific virulence traits contribute to the environmental persistence and pathogenic potential of *A. baumannii* ST25 isolates.

## INTRODUCTION

*Acinetobacter baumannii* is an opportunistic pathogen responsible for healthcare-associated infections occurring worldwide. Less frequently, *A. baumannii* has been associated with community-acquired infection in fragile patients (1). These infections are difficult to treat because the majority of *A. baumannii* isolates are resistant to at least three different classes of antimicrobials, including carbapenems, and retain susceptibility to colistin only (1). Because of this, the World Health Organization classifies carbapenem-resistant *A. baumannii* as a “critical” pathogen among the antibiotic-resistant bacteria of global priority (2).

While the hospital environment has been considered the primary reservoir of *A. baumannii* (1, 3), additional sources such as animals, food, poultry livestock, soil, plants, freshwater, sludge, and wastewater were identified (4–10).

The global epidemiology of *A. baumannii* isolates has shown that the population structure of the species was characterized by the selection of genetically distinct clonal lineages, three international clonal lineages (ICL) I, II, and III, and up to eight additional epidemic clonal lineages (11–13). The ST25 *A. baumannii* clone, according to Pasteur’s MLST scheme (14), emerged as a globally distributed multidrug-resistant lineage responsible for epidemics in Europe, South America, Africa, and Asia (15–17). Isolates belonging to the ST25 lineage have also been reported from infections in animals, in particular urinary tract infections, and in veterinary clinics (5, 8, 10). The phylogenetic analysis of ST25 *A. baumannii* lineage identified four clades (CI–CIV) with different geographical distribution. In particular, isolates belonging to CI and CIII clades originated from South America, whereas those included in CII and CIV clades have global geographical origin (5). ST25 *A. baumannii* was also isolated from chick-box papers of turkey chicks (6). Further studies demonstrated that ST25 *A. baumannii* isolates possess specific capsular polysaccharide loci (KL) and lipooligosaccharide outer core locus (OCL) types and show a high variability in these virulence determinants (18, 19).

In addition to resistance to a broad range of antimicrobials, *A. baumannii* epidemic clonal lineages possessed virulence-related traits, such as biofilm growth, adherence to host epithelial cells, resistance to desiccation, resistance to oxidative stress, and the ability to kill *Galleria mellonella* larvae, which contributed to replication into infected hosts and persistence in contaminated environments (3, 20). Since knowledge on specific virulence traits of the ST25 lineage is lacking, the present study aimed to (i) update the phylogenetic analysis and KL and OCL typing of ST25 *A. baumannii*; (ii) identify the genotypic virulence profiles of 203 ST25 *A. baumannii* genomes from human, animal, and environmental isolates; (iii) analyze phenotypic virulence-related and stress-related traits of 40 representative ST25 *A. baumannii* isolates; and (iv) correlate genotypic virulence profiles with phenotypic virulence-related and stress-related traits of ST25 *A. baumannii* isolates.

## MATERIALS AND METHODS

### Database of genomes and phylogeny

A collection of 203 *A*. *baumannii* genomes, all assigned to ST25 sequence type according to the Pasteur MLST scheme (14), was assembled from (i) the data set (*n* = 148) previously described by Lupo et al. (5) and (ii) genomes (*n* = 55) manually selected from the PubMLST database (21) up to 1 May 2024. The characteristics of the genomes were included in Table S1 and are available as a public project: pubmlst.org/bigsdb?db=pubmlst_abaumannii_isolates&page=project&project_id=23. The reference strains *A. baumannii* AYE (ST1), ATCC 19606 (ST52), ATCC 17978 (ST437), and ACICU (ST2) were also included in the study (22–25). The core-genome analysis was undertaken based on 2,386 conserved genes defined within the *A. baumannii* cgMLST scheme (https://github.com/bvalot/pyMLST). Phylogenetic reconstruction was carried out using MrBayes version 3.2.7, applying the GTR + G  +  I nucleotide substitution model (26). The resulting phylogenetic tree was midpoint-rooted and visualized using Interactive Tree of Life (iTOL) version 6 (27).

### *In silico* typing of virulence factors associated with ST25 isolates

A comprehensive screening of virulence-associated genes was performed across all 203 ST25 *A. baumannii* genomes. We used the pubMLST software (21) to develop *A. baumannii*-specific virulence genes database. Our curated list comprised 127 genes, selected from three main sources: the ResFinder database (six genes belonging to effector delivery system or enzymes categories), the PubMLST virulence gene collection (111 genes belonging to biofilm, adherence, EPs, quorum sensing, metabolism/nutrition, or immune modulation categories), biofilm-associated proteins (Baps)1-2-3 as described by De Gregorio et al. (28), and genes involved in iron acquisition and metabolism previously characterized by Artuso et al. (29). The virulence genes were grouped and classified in functional categories and are available on pubMLST (https://pubmlst.org/bigsdb?db=pubmlst_abaumannii_isolates&page=plugin&name=GenePresence). A threshold identity of 80%, a minimum length of 80% matches, and a coverage value of more than 90% were the values considered for establishing the presence of each virulence gene.

To further characterize the surface polysaccharide loci of each ST25 *A. baumannii* genome, KL and OCL typing was performed using Kaptive 2.0.4 (18, 19) and the CAGECAT platform (https://cagecat.bioinformatics.nl/)

To assess the pangenome characteristics, PPanGGOLiN v1.1.136 (30) was used with default settings to classify gene families into persistent, shell, and cloud partitions. As input, genome annotations in GFF3 format were generated using Bakta v1.8.2 (31), also executed with default parameters.

### Bacterial isolates and growth conditions

Forty ST25 representative isolates available from ANSES-Laboratory Lyon and Public Health-Laboratory Naples, and the reference strains *A. baumannii* ACICU, AYE, ATCC 19606, and ATCC 17978 (5, 12, 15, 22–24) were used in phenotypic assays to analyze their virulence ability. To investigate the role of EPs on virulence features of ST25 *A. baumannii*, a set of isogenic mutants derived from the *A. baumannii* ATCC 19606 wild-type strain was used. These included gene deletions targeting *adeB* and *adeJ* (belonging to the RND, Resistance-Nodulation-Division family), *aceI* (PACE, Proteobacterial Antimicrobial Compound family), and *amvA* (MFS, Major Facilitator Superfamily), as previously described by Migliaccio et al. (32). All isolates were cultured under aerobic conditions at 37°C in Luria-Bertani (LB) broth/agar. LB broth/agar, phosphate-buffered saline (PBS) 1×, and tryptic soy broth (TSB) were used to perform biofilm formation, oxidative stress resistance, serum resistance, desiccation resistance, and *Galleria mellonella* larvae virulence assays. The chemical reagents were purchased from Sigma-Aldrich (Sigma, Milan, Italy).

### Biofilm assays

Biofilm formation was assessed using the crystal violet (CV) staining assay as previously described (33, 34) and estimated according to the European Committee for Antimicrobial Susceptibility Testing (35). Bacterial cell suspensions were adjusted to 0.5 McFarland using a BD PhoenixSpec nephelometer after an overnight (O/N) growth in TSB. Subsequently, the bacterial cells were diluted to a final culture density of approximately 1 × 10^6^ colony-forming units (CFU)/mL in TSB and were transferred into a 96-well flat-bottomed polystyrene microtiter plate. The microplates were incubated at 37°C for 24 h. The culture supernatants were gently discarded, the wells were washed twice with PBS pH 7.4, and the biofilms were stained with 200 μL of 0.1% CV for 20 min. The wells were washed twice with PBS, and the linked CV was eluted with ethanol. The absorbance was measured at 595 nm using a microplate reader (Bio-Rad Laboratories S.r.l.). The OD_595_/OD_600_ ratio was used to normalize the amount of biofilm formed versus growth.

### *G. mellonella* larvae virulence assays

*A. baumannii* ST25*,* ATCC 19606, ATCC 17978, ACICU, and AYE strains were grown in LB until late exponential phase (0.4–0.5 OD_600_). Cells were collected by centrifugation and suspended in PBS. *G. mellonella* larvae were purchased by Insect Novel Ecologic Food S.r.l, Padua, Italy. Serial 10-fold dilutions of bacterial cell suspensions in PBS 1× were injected into *G. mellonella* larvae as described previously (36). Ten larvae were infected with each infecting dose (from ~1  ×  10^5^ to ~1  ×  10^8^ CFU/mL), and 10 larvae were injected with PBS as a negative control. Larvae were incubated at 37°C for 96 h to monitor the survival. Each isolate was tested in three independent experiments. Dose-dependent survival curves and 50% (LD_50_) and 90% (LD_90_) lethal doses were determined using the GraphPad Prism software as previously described (36). The results obtained were plotted to generate Kaplan-Meier survival curves (GraphPad Prism version 10.0).

### Resistance to desiccation assays

The desiccation assay was performed as previously described (20). Overnight LB cultures were centrifuged at 12,000 × *g* for 5 min in a microcentrifuge. The cell pellet was washed twice with PBS and suspended to 1 OD_600_. Twenty microliters of each suspension was deposited onto a glass cover slip to produce an inoculum of 1 × 10^7^ CFU/mL. The coverslip was kept at 30% + 5% relative humidity by the presence of a saturated CaCl_2_ in an uncovered Petri dish and stored at room temperature in an air-tight transparent plastic box (17 × 11  ×  5.5 cm) for up to 60 days. Viable cell counts were determined by detaching and seeding on LB agar and further incubation at 37°C O/N.

### Serum resistance assays

Serum resistance assays were performed as described previously by Shin et al. (37) with minor modifications. Overnight bacterial cultures were diluted 1:100 into 10 mL of fresh LB medium and incubated until the bacterial suspension reached 0.5 OD_600_. Then, 1 mL aliquot of the culture was washed with PBS and resuspended in the same quantity. Subsequently, 100 μL of the bacterial suspension was added in 96-multiwell and mixed with bovine serum diluted to 20%; the inactivated serum at 56°C for 45 min was also used. After mixing, the serum-bacteria suspensions were incubated at 37°C for 60 min. To calculate the serum bactericidal effect, a 100-µL aliquot was taken from each suspension, serially diluted, and plated on LB agar. The serum bactericidal effect was expressed as CFU/mL of viable cells.

### Oxidative stress tolerance

Oxidative stress tolerance was tested by exposing 0.1 OD_600_ of O/N growth of representative clades *A. baumannii* ST25 strains, ATCC19606, ATCC17978, AYE, ACICU, and EPs mutant strains to 150 µM H_2_O_2_ for 9 h at 37°C under shaking. Bacteria were exposed to 50–200 µM H_2_O_2_. These concentrations were obtained by diluting in PBS from the stock solution (9.8 × 10^6^ µM at pH 4.0). To calculate the H_2_O_2_ effect, a 100 µL aliquot was taken each hour, serially diluted, and plated on LB agar, incubated at 37 °C O/N (38).

### RNA purification and real-time quantitative PCR

*A. baumannii* strains were grown as for the serum assay or oxidative stress assays. The bacterial cells were washed with PBS, and total RNA was isolated via TRIzol reagent (Qiagen, Milan, Italy). The cDNAs were synthesized using QuantiTect Reverse Transcription Kit (Qiagen, Milan, Italy), according to the manufacturer’s protocol. Real-time quantitative PCR (RT-qPCR) assays were performed using SYBR Green master mix (Applied Biosystems) (36). The *rpoB* housekeeping gene was used to normalize the expression of target genes (39), and the fold changes of gene expression levels were calculated using the 2^–ΔΔct^ method (40). All experiments were performed three times in triplicate. The primers used in the RT-qPCR experiments were previously reported (32).

### Statistical analysis

All statistical analyses were conducted using GraphPad Prism version 10.0 (GraphPad Software, San Diego, CA, USA). Differences among multiple groups were assessed using two-way analysis of variance (ANOVA), followed by the Bonferroni post hoc test to correct for multiple comparisons. Statistical significance was defined as *P* < 0.05, *P*<0.01, and *P*<0.001. Two-way ANOVA and Tukey test *P*-values were utilized for statistical analysis of biofilm experiments. Spearman’s rank correlation coefficient (ρ) was used to assess the relationship between the expression levels of efflux pump (EP) genes and the corresponding phenotypes of EP mutants, in comparison to the ST25 isolates. This non-parametric test was selected due to the ordinal nature of the expression data and the potential lack of normal distribution. Correlation analyses were performed to investigate potential associations between KL/OCL genotypes and phenotypic traits of virulence and stress resistance. Specifically, the contingency tables were constructed for each phenotype, and chi-square (χ²) tests were performed. Cramer’s V coefficient was calculated to quantify the strength of the associations based on degrees of freedom (df), and *P*-values were used to assess statistical significance (*P* < 0.05) (41). All data were presented as means ± standard deviation. The number of replicates was two or three times.

## RESULTS

### Phylogenomic structure and KL and OCL typing of ST25 *A. baumannii* clonal lineage

**Fig 1:**
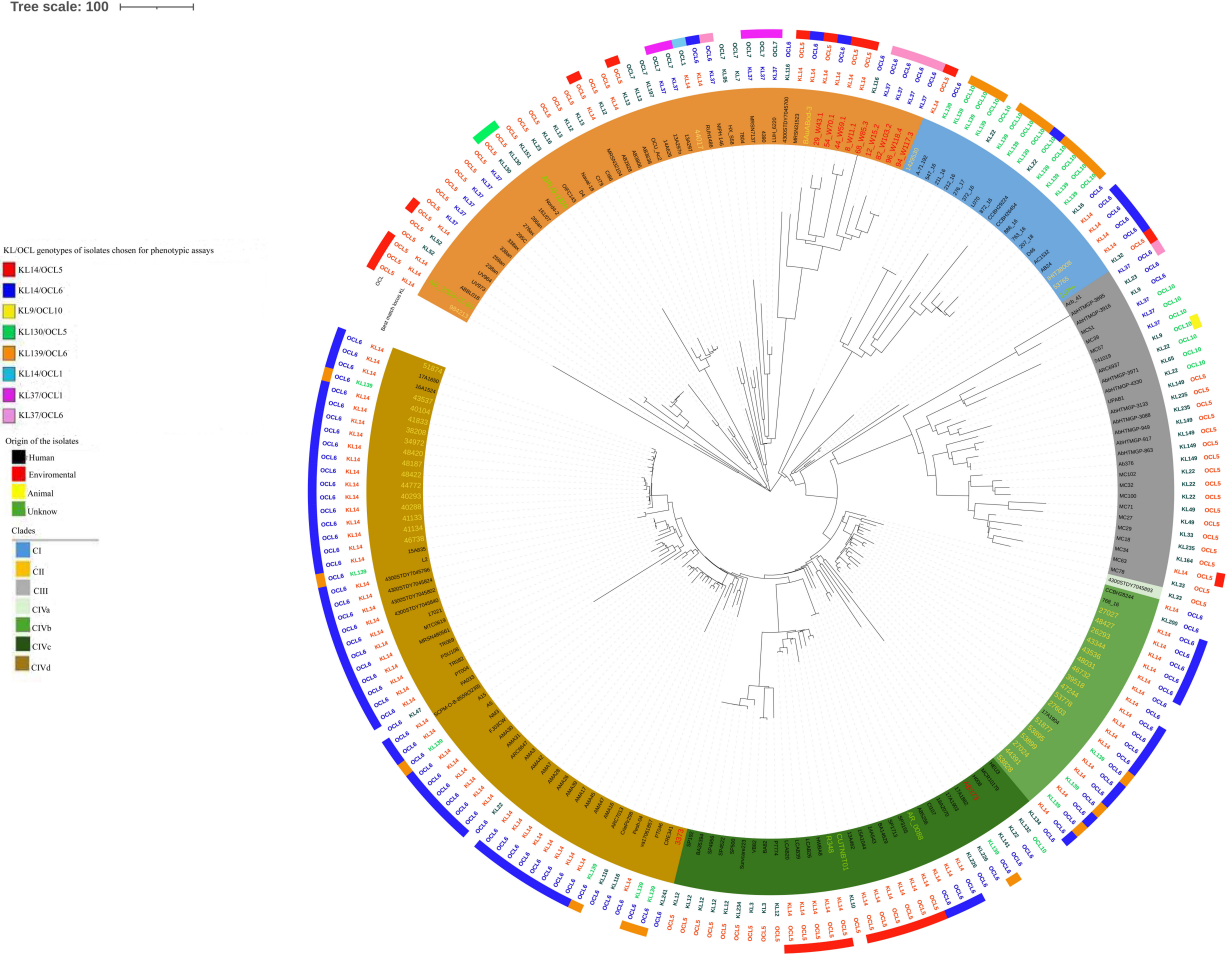
cgMLST-based phylogeny of 203 *A*. *baumannii* ST25 genomes. The phylogenetic tree is annotated with concentric colored rings as follows: the outermost ring indicates the KL types, the second ring from the outside represents OCL types, and the innermost ring indicates clades and subclades. Distribution of KL/OCL types corresponding to the KL/OCL genotypes of isolates selected for phenotypic assays, indicated by colored rectangles outside the tree. The colors of the isolates specify the origin of isolation: 150 isolates from humans in black, 36 from animals in yellow, and 10 from environmental sources in red, 7 isolates of unknown origin in green.

The core-genome MLST analysis of 203 *A*. *baumannii* ST25 genomes distinct clades: CI (20 genomes), CII (48 genomes), CIII (27 genomes), and CIV (108 genomes) (Fig. 1). Clade IV was further subdivided into subclades IVa (1 genome), IVb (20 genomes), IVc (31 genomes), and IVd (56 genomes) (Fig. 1).

**Fig 2:**
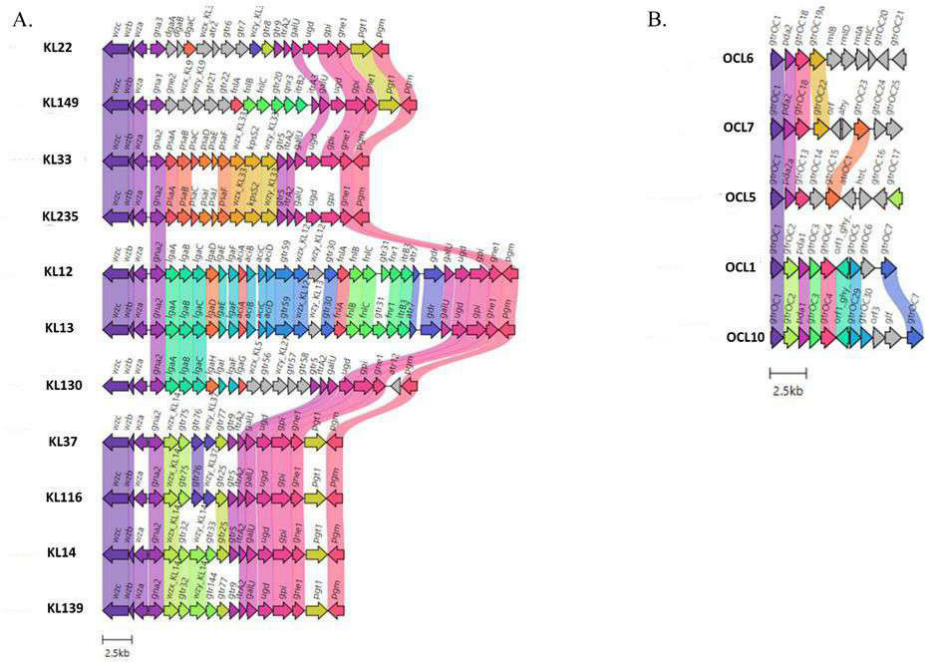
Comparative analysis of representative KLs (**A**) and OCLs (**B**). The colors indicating the alignment of the KL/OCL loci use an identity threshold of 0.98.

In addition to the population structure analysis, this study also assessed the capsular KL and OCL profiles of ST25 *A. baumannii* genomes. Each genome was characterized by a specific KL and OCL combination, adding resolution to the genomic diversity within the ST25 lineage (Table S1). KL14 was the predominant capsular locus, found in 47% of genomes assigned to clades CIV (80%) and CII (20%) (Fig. 1); KL139 and KL37 were found in 10.3% and 9.8% of genomes, respectively. OCL6 was the most frequent OCL type (50%), followed by OCL5 (35%) and OCL10 (9.8%). These isolates originated from diverse sources, including humans (150 genomes), animals (36 genomes), the environment (10 genomes), and unknown (7 genomes). The human-derived isolates were distributed across all clades and displayed considerable heterogeneity in KL and OCL types (Table S1; Fig. 1). Instead, animal-derived isolates belonged to clades CII, CIVb, and CIVd and carried the KL14/OCL6 combination (33 out of 36 genomes). This KL14/OCL6 combination was especially predominant in CIVb (*n*=16/20) and CIVd (*n*=44/56) sub-clades independently of the host (Fig. 1). Environmental isolates belonged to clades CII (9 genomes) and CIVc (1 genome) and were associated with KL14/OCL5 (4 out of 9 genomes), KL37/OCL6 (4 out of 9 genomes), and KL10/OCL6 (1 out of 1 genome) (Table S1; Fig. 1). The most common KL types shared identical genes at the 5′ and the 3′ of the locus (Fig. 2A). In detail, capsular export genes *wzc* and *wzb*, but not *wza*, showed 100% nucleotide identity and were found at the 5′ of KL22, KL129, KL33, KL116, KL37, KL14, and KL139, while identical *pgm* and *gne1* genes were found at the 3′ of all prevalent loci (Fig. 2A).

Contrarily, the OCL loci showed relatively higher structural conservation, particularly among OCL10, OCL5, and OCL6 (Fig. 2B). The glycosyltransferases encoding gene, *gtrOC1,* was found identical in all OCL types, while *gtrOC2*, *gtrOC3,* and *gtrcOC4* were identical in OCL1 and OCL10. Also, the identical polysaccharide deacetylase *pda2* gene was found in OCL6 and OCL7, a similar polysaccharide deacetylase *pda2a* gene was found to be associated with OCL7 and OCL5, and an identical *pda1* gene was found in OCL1 and OCL10 (Fig. 2B).

### *In silico* analysis of virulence genes in *A. baumannii* ST25 genomes

Among the adherence category, distinct *pil-* and *fim-* genes were present in the 203 ST25 *A. baumannii* genomes analyzed, while *ata*, *pilA,* and *pilC* were absent (Table S2). In the effector delivery system category, all genes of T2SS and some genes of the T6SS subcategories were present in ST25 genomes. In the biofilm category, the genes belonging to Poly-β−1,6-N-acetyl-D-glucosamine, quorum sensing, *csu* fimbriae, and *bap-3* (type-3 biofilm-associated protein) encoding genes were present in all ST25 genomes. In the metabolism/nutrition category, the eight genes belonging to the *hemO* cluster subcategory were present in all ST25 genomes, while the *bauA* gene of acinetobactin subcategory was present in 7% of ST25 genomes. The *adeA* and *adeB* genes encoding the AdeABC EP system and *adeI, adeJ,* and *adeK* of the AdeIJK efflux system, along with *adeN*, *adeL, adeR,* and *adeS* transcriptional regulators, were present in all ST25 genomes. Among the regulation categories, the Bfmrs regulatory system is present in all ST25 genomes. Among the exoenzyme category, *plcD, plc2, plc1* exotoxin genes, and *cpaA* gene are present in all ST25 genomes (Table S2).

### ST25 *A. baumannii* pangenome analysis

Pangenome analysis of the 203 ST25 *A. baumannii* genomes generated a K3 gene family partition into persistent, cloud, and shell, based on their frequency across genomes (Fig. S1). The persistent group, comprising 3,084 genes present in all 203 genomes, was considered the core genome. The cloud (3,597) and shell (2,118) genes were identified in 1–8 and 8–198 genomes, respectively, and were considered the accessory genome (Table S3).

We carefully analyzed the shell partition, which includes moderately conserved genes across the genomes and could potentially reflect clade-specific traits. Among shell gene families, the genes encoding Fe-S hydro-lyase tartrate dehydratase beta-type catalytic domain-containing protein, tartrate dehydratase, a putative tartrate transporter, and an HTH-type transcriptional regulator DmlR were found only in CII clade genomes (or CII clade-specific) (Table S3). Also, gene families associated with phage-related functions and hypothetical proteins, including DUF domain-containing proteins and various bacteriophage tail and head components, were identified in the 129 genomes belonging to CIV (108/108 genomes) (or CIV semi-clade-specific), CII (10/53 genomes), and CIII (11/27 genomes) (Fig. S1; Table S3).

### Virulence-related traits analyses of ST25 *A. baumannii*

Based on the *in silico* results, a subset of 40 ST25 *A. baumannii* isolates belonging to different KL/OCL genotypes was included for *in vivo* and *in vitro* analyses of the most common virulence traits in *A. baumannii*. The selected ST25 *A. baumannii* isolates were assigned to the following genotypic capsular profiles: KL14/OCL6 (one isolate) assigned to clade CI, KL14/OCL6 (two isolates), KL14/OCL1 (1), KL37/OCL6 (1), KL37/OCL7 (1), and KL130/OCL5 (1) for isolates assigned to clade CII; KL9/OCL10 for clade CIII (one isolate); KL14/OCL6 (16) and KL139/OCL6 (1) for subclade CIVb; KL14/OCL5 (3) for subclade CIVc; and KL14/OCL6 (14) for subclade CIVd (Tables S1 and S4).

### Virulence profiles of ST25 *A. baumannii* strains in *G. mellonella* larvae

The virulence potential of ST25 *A. baumannii* selected isolates was analyzed using the *Galleria mellonella* infection model and results compared with those of AYE, ACICU, and ATCC 19606 *A*. *baumannii* strains (Table 1; Fig. S2). Dose-dependent LD_50_ and LD_90_ values in *G. mellonella* larvae showed distinct virulence profiles among ST25 *A. baumannii* strains belonging to the different clades and KL/OCL types. In particular, CIVd and CIVb strains showed a high infectivity with LD_50_ and LD_90_ values ranging from 1 × 10^6^ to 4 × 10^7^ CFU/mL and 2 × 10^6^ to 6 × 10^7^ CFU/mL, respectively (Table 1). In contrast, LD_50_ and LD_90_ of CI, CII, CIII, and CIVc strains were 3.33- and 5-fold higher than LD_50_ and LD_90_ of CIVd and CIVb strains (*P* < 0.01). The virulence profiles of CIVd and CIVb strains were similar to those of AYE and ATCC 19606 strains, which displayed LD_50_ and LD_90_ values ranging from 1 × 10^7^ CFU/mL to 4 × 10^7^ CFU/mL, while they were lower than that of the ACICU strain, which showed LD_50_ and LD_90_ values of 1 × 10^5^ CFU/mL and 9 × 10^5^ CFU/mL, respectively. Moreover, a significant association was found between KL/OCL genotypes and virulence in *G. mellonella*, with nearly all (*n* = 22/29) KL14/OCL6 genotypes being highly virulent (Cramer’s V = 0.94, χ² = 35.4, df = 7, *P* = 9.4 × 10⁻⁶).

**TABLE 1:** Lethal doses 90% (LD_90_) and 50% (LD_50_) in *G. mellonella* larvae for the indicated *A. baumannii* strains^*a*^

Strain	LD90	LD50	R^2	Genotype	
53765	1,9x10^6	1x10^7	0,877	CI ST25	KL14/OCL6
4390	1,5x10^8	2,6x10^7	0,785	CII ST25	KL37/OCL7
44017	1,9x10^6	1x10^7	0,777	CII ST25	KL14/OCL1
161/07	2x10^8	1x10^7	0,845	CII ST25	KL130/OCL5
NIPH146	4x10^8	1x10^8	0,775	CII ST25	KL37/OCL6
RUH1486	1x10^7	5x10^6	0,821	CII ST25	KL14/OCL6
741019	1x10^8	1,2x10^7	0,789	CIII ST25	KL9/OCL10
26293	1x10^7	9x10^6	0.825	CIVb ST25	KL14/OCL6
27024	2,2x10^6	1x10^6	0,881	CIVb ST25	KL14/OCL6
27027	2,1x10^6	1x10^6	0,777	CIVb ST25	KL14/OCL6
27603	4x10^7	1x10^7	0,888	CIVb ST25	KL14/OCL6
39518	6x10^5	1x10^6	0,744	CIVb ST25	KL14/OCL6
43344	2x10^6	1x10^6	0,912	CIVb ST25	KL14/OCL6
43536	1,5x10^6	9x10^6	0,778	CIVb ST25	KL14/OCL6
44391	9x10^7	2x10^7	0,699	CIVb ST25	KL139/OCL6
46732	1,5x10^6	1x10^7	0,811	CIVb ST25	KL14/OCL6
47244	4x10^6	1x10^6	0,712	CIVb ST25	KL14/OCL6
48031	1x10^8	6x10^7	0,846	CIVb ST25	KL14/OCL6
48427	1x10^7	8x10^6	0,835	CIVb ST25	KL14/OCL6
51877	1x10^7	1x10^6	0,745	CIVb ST25	KL14/OCL6
53778	2x10^6	1x10^6	0,755	CIVb ST25	KL14/OCL6
53828	1x10^7	5x10^6	0,788	CIVb ST25	KL14/OCL6
53895	9x10^7	4x10^7	0,689	CIVb ST25	KL14/OCL6
53899	1,7x10^6	1x10^7	0,899	CIVb ST25	KL139/OCL6
14A543	1x10^8	2x10^7	0,854	CIVc ST25	KL14/OCL5
13A462	1x10^8	3x10^7	0,875	CIVc ST25	KL14/OCL5
15A1044	1x10^8	1x10^7	0,799	CIVc ST25	KL14/OCL5
34792	1x10^8	9x10^7	0,775	CIVd ST25	KL14/OCL6
40104	5x10^6	1x10^6	0,888	CIVd ST25	KL14/OCL6
40293	4x10^7	1x10^7	0,754	CIVd ST25	KL14/OCL6
41133	2x10^6	1x10^6	0,887	CIVd ST25	KL14/OCL6
41833	4x10^6	1x10^6	0,789	CIVd ST25	KL14/OCL6
43537	9x10^6	6x10^6	0,845	CIVd ST25	KL139/OCL6
46738	1x10^7	1x10^6	0,689	CIVd ST25	KL14/OCL6
44772	6x10^6	1x10^6	0,778	CIVd ST25	KL14/OCL6
48420	1x10^7	9x10^6	0,884	CIVd ST25	KL14/OCL6
48422	6x10^7	1x10^7	0,845	CIVd ST25	KL14/OCL6
51874	2x10^6	1x10^6	0,788	CIVd ST25	KL14/OCL6
16A1524	1x10^7	6x10^6	0,811	CIVd ST25	KL14/OCL6
17A1650	1x10^7	8x10^6	0,847	CIVd ST25	KL14/OCL6
AYE	4x10^7	2x10^7	0,789	ST1	KL1/OCL1
ACICU	9x10^5	1x10^5	0,845	ST2	KL2/OCL1
ATCC 19606	3x10^7	1x10^7	0,874	ST52	KL3/OCL1

^*a*^ LD_50_ and LD_90_ are expressed as the number of viable cells.

### Biofilm formation ability of ST25 *A. baumannii*

**Fig 3:**
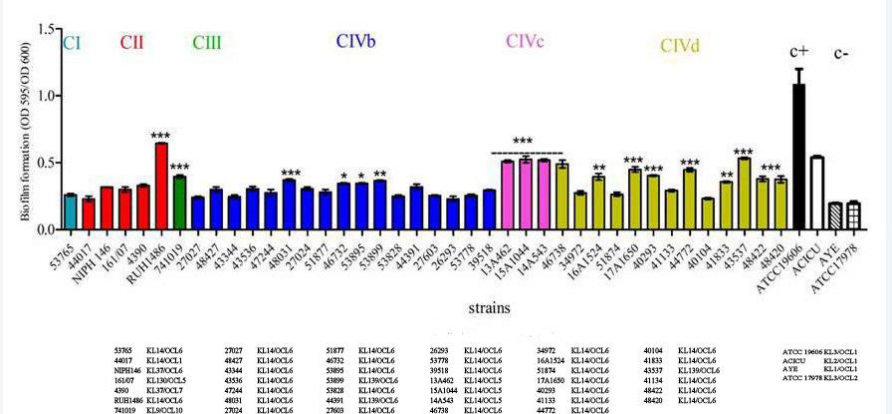
Biofilm formation of ST25 *A. baumannii* strains. The data were obtained from three independent experiments in which each isolate was tested in triplicate. The significance of the differences was calculated using two-way ANOVA (*P-values < 0.5 , **P-values <0.01, and ***P-values <0.001). The isolates’ names and their respective KL/OCL genotypes are shown at the bottom of the graph.

The biofilm growth of ST25 *A. baumannii* strains was analyzed using the ATCC 19606 strain as a positive control (42), and AYE and ATCC 17978 as negative controls (15, 43) (Fig. 3). Among the 40 ST25 isolates, 19 (1/1 CI, 1/5 CII, 1/1 CIII, 4/17 CIVb, 3/3 CIVc, and 9/14 CIVd isolates) showed 1.5- to 2.5-fold higher biofilm formation than AYE and ATCC17978 controls, but less biofilm formation than ATCC19606 (Fig. 3) and were classified as intermediate biofilm producers (43).

Our data showed a high degree of heterogeneity in biofilm formation of ST25 *A. baumannii* isolates assigned to different clades, while identifying a correlation between specific KL/OCL genotypes and biofilm formation among the 40 ST25 A. *baumannii* isolates. In particular, biofilm production was strongly associated with KL14/OCL5 and KL139/OCL6 genotypes, while KL14/OCL6 isolates were biofilm-non-producer (Cramer’s V = 0.85, χ² = 28.7, df = 7, *P* = 0.00016) (Fig. 3; Table S4).

### Resistance to desiccation of ST25 *A. baumannii* strains

**Fig 4:**
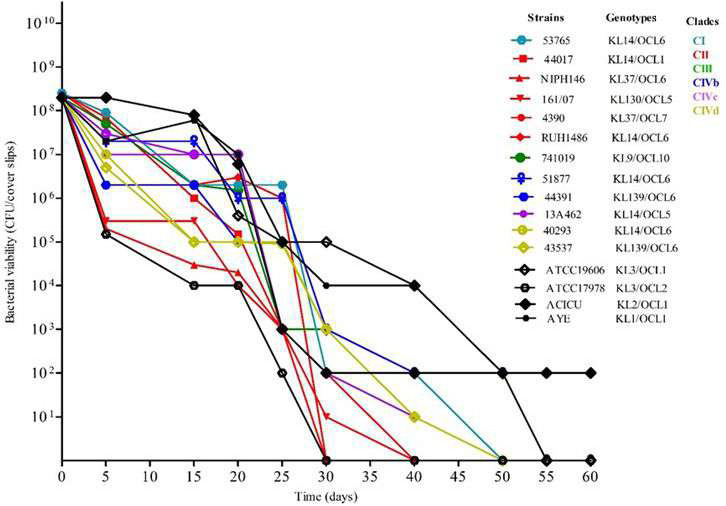
Desiccation resistance of ST25 *A. baumannii* strains. The viable cells (CFU/mL) were determined for each isolate after incubation on glass slips in a 30% ± 5% humidity environment. The data were obtained from three independent experiments in which each isolate was tested in triplicate.

The ability of ST25 *A. baumannii* strains to survive under desiccating conditions on abiotic surfaces was evaluated and compared with that of ATCC 19606, AYE, ACICU, ATCC 17978 *A*. *baumannii* strains, and ATCC 19606 EPs mutant strains. Strains from clades CI, CII, and CIII exhibited notable variability in survival, with several CII isolates, such as 44017 and 4390, retaining viability up to 30 days, while others declined more rapidly (Fig. 4). Interestingly, the CIII isolate 741019 demonstrated higher resistance to desiccation than CI and CII strains and maintained viability beyond 50 days (Fig. 4).

Clade CIVb strains showed more consistent and prolonged survival, with many isolates retaining detectable CFUs up to 50–60 days. Similarly, isolates from clade CIVd showed resistance to desiccation, with several strains, including 16A1524 and 40293, maintaining viability well beyond 40 days. Similarly, clade CIVc strains displayed survival until 50 days (Fig. 4). Collectively, these findings highlighted considerable variability in desiccation tolerance across *A. baumannii* clades and strains. ST25 *A. baumannii* strains showed similar desiccation resistance compared with ATCC 19606, AYE, ACICU, and higher desiccation resistance compared with ATCC 17978 (Fig. 4). In particular, AYE and ACICU strains showed the highest survival at 60 days, with 10^4^ and 10^2^ CFU/mL, respectively. In contrast, ATCC 17978 showed a rapid decline, with no viable cells detected beyond day 20 (Fig. 4). Also, ST25 strains assigned to KL14/OCL6, KL139/OCL6, and genotypes consistently displayed long survival to desiccation, while KL9/OCL10 was susceptible (χ² = 40.0, df = 7, *P* = 1.26 × 10⁻⁶; V = 1.00) (Fig. 4; Table S4).

To investigate the contribution of EPs to desiccation survival in *A. baumannii*, the ability of ATCC 19606 *A. baumannii* and ATCC 19606 EPs mutant strains to survive under desiccating conditions was investigated. The parental strain ATCC 19606 survived up to 60 days, whereas EP mutants displayed differential desiccation tolerance (Fig. S3). The *ΔadeB* strain notably showed impaired long-term survival, with CFUs dropping sharply after day 50. The *Δamva, Δacel,* and *ΔadeJ* strains exhibited markedly reduced survival in CFU/mL terms, compared with wild type or *ΔadeB,* becoming undetectable around day 50 (Fig. S3).

### Oxidative stress tolerance and expression of EP genes under oxidative stress in ST25 *A. baumannii*

**Fig 5:**
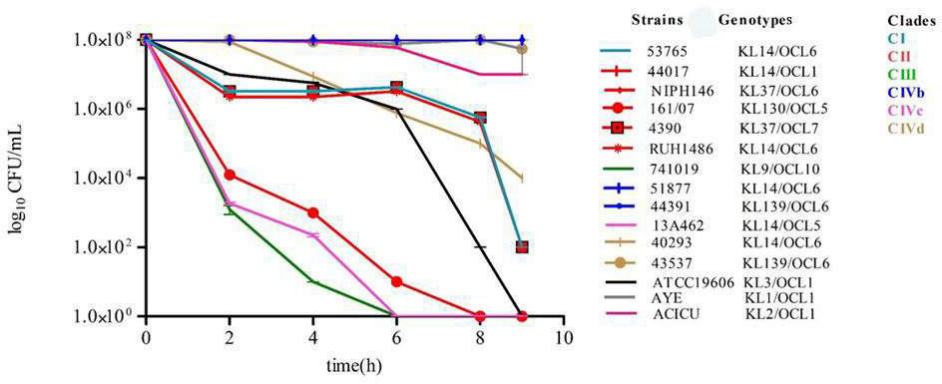
Oxidative stress tolerance of ST25 *A. baumannii* strains. The viable cells (CFU/mL) were determined for each isolate after 0–9 h of 150 µM H₂O₂ exposure. The data were obtained from three independent experiments in which each isolate was tested in triplicate.

We evaluated the ability of ST25 *A. baumannii* strains to tolerate oxidative stress following exposure to 150 µM H_2_O_2_. Strains 51877 and 44391, belonging to CIVb, displayed strong resistance to 150 µM H_2_O_2_, maintaining high CFU levels (10^8^ CFU/mL) over 8 h. In contrast, 741019 of CIII, 13A462 strains belonging to CIVc showed higher sensitivity than CIVb strains and rapidly declined below the detection limit within 2–6 h. Furthermore, CI, CII, and CIVd strains showed 10^7^ CFU/mL until 6 h, and then the viability decreased to 10^4^–10^2^ CFU/mL (Fig. 5). These data indicated substantial heterogeneity among ST25 *A. baumannii* isolates in oxidative stress response. Similarly to desiccation resistance, ST25 *A. baumannii* strains assigned to KL14/OCL6 and KL139/OCL6 genotypes consistently displayed high resistance to H₂O₂, while those assigned to KL14/OCL5, KL130/OCL5, and KL9/OCL10 genotypes were generally susceptible (χ² = 40.0, df = 7, *P* = 1.26 × 10⁻⁶; V = 1.00) (Fig. 5; Table S4).

**Fig 6:**
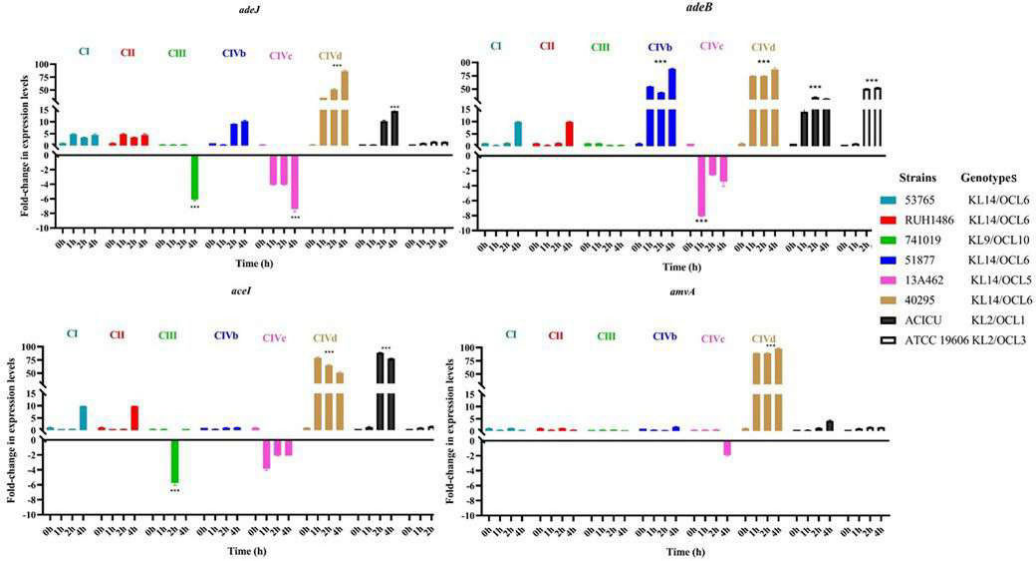
Fold change in expression levels of *adeJ, adeB, aceI,* and *amvA* EPS genes in ST25 *A. baumannii* after 0, 1, 2, and 4 h of H_2_O_2_ exposure. The significance of the differences was calculated using two-way ANOVA (**P-values <0.01, ***P-values < 0.001).

To study the role of EPs in oxidative stress tolerance in ST25 *A. baumannii*, ATCC 19606 *A*. *baumannii,* and ATCC 19606, EP mutant strains were included. The targeted deletions in EP genes (*∆aceI, ∆amvA, ∆adeJ,* and *∆adeB*) impaired H₂O₂ survival compared to the wild-type ATCC19606. Notably, *∆adeJ* and *∆adeB* strains were highly susceptible, counting 10^4^ or 10^2^ CFU/mL cells viable after 2 h of exposure (Fig. S4). These results suggested that these efflux systems contributed directly or indirectly to oxidative stress protection, potentially by exporting toxic compounds or maintaining redox balance. The expression of EPs genes in ST25 *A. baumannii* isolates was thus assessed under exposure to 150 mM H₂O₂. All Ep gene expressions were significantly upregulated at 1, 2, and 4 h in isolate 40293 belonging to CIVd; *adeJ* and *adeB* genes were significantly upregulated at 2 and 4 h in isolate 51788 belonging to CIVb. In contrast, *aceI* and *amvA* were downregulated but not significantly in 13A462 assigned to CIVc; *adeB* and *adeJ* genes were significantly downregulated, and *aceI* gene expression was downregulated but not significantly in 13A462 strain belonging to CIVc (Fig. 6).

The expression profiles and mutant phenotypes suggested a link between the upregulation of *adeB*, *adeJ,* and *aceI* gene expression and the ability of ST25 *A. baumannii* isolates to tolerate oxidative stress. Notably, the high tolerance to oxidative stress of CIVd and CIVb isolates correlated with *adeB* and *adeJ* overexpression (Spearman *p-value* corresponding to 0.0173 and 0.0166, respectively).

### Serum resistance and expression of genes encoding efflux systems under serum exposure in ST25 *A. baumannii*

**Fig 7:**
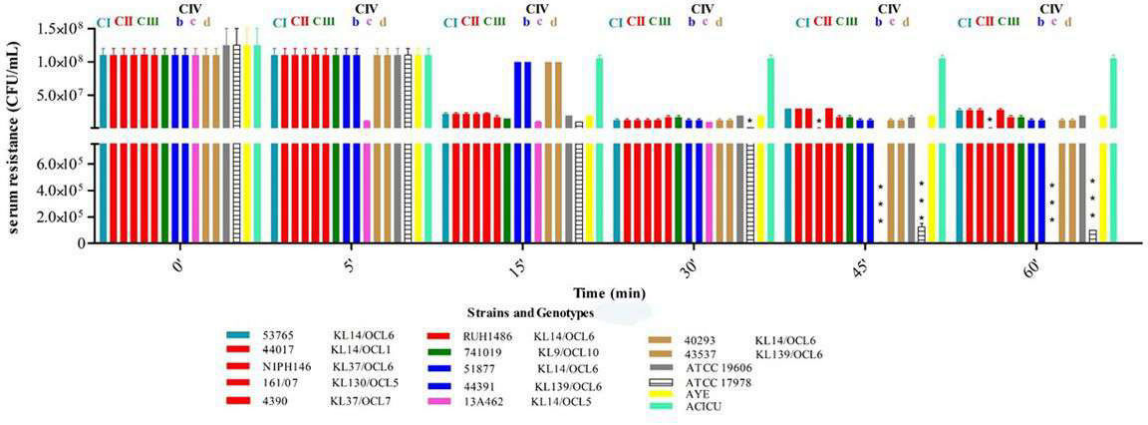
Serum resistance of ST25 *A. baumannii*, ST2 ACICU, ST1 AYE, and ST52 ATCC 19606. The viable cells (CFU/mL) were determined for each isolate following a 5- to 60-min incubation in 20% activated serum and normalized using values obtained from incubation with heat-inactivated. The data were obtained from three independent experiments in which each isolate was tested in triplicate. ***P-values <0.001.

The majority of ST25 *A. baumannii* isolates within CI, CII, CIII, CIVb, and CIVd exhibited high levels of serum resistance, maintaining bacterial viability (10^7^–10^8^ CFU/mL) even after 60 min of exposure. In contrast, isolates belonging to CIVc showed a significant decline in viability over time, even after 45-min exposure to serum. Furthermore, ST25 *A. baumannii* isolates assigned to KL14/OCL6 and KL139/OCL6 consistently displayed high resistance to serum treatment, while KL14/OCL5 and KL9/OCL10 were more sensitive (χ² = 40.0, df = 7, *P* = 1.26 × 10⁻⁶; V = 1.00) (Fig. 7; Table S4).

**Fig 8:**
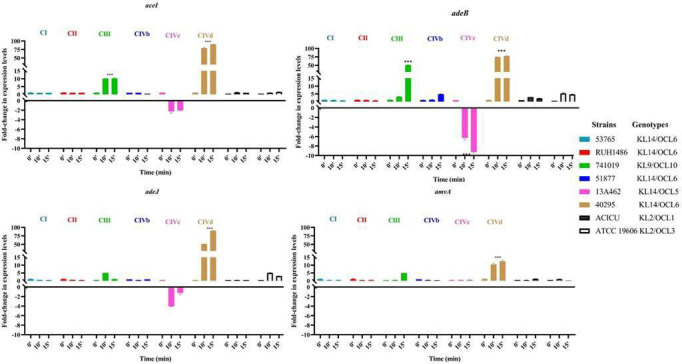
Fold change in expression levels of *adeJ, adeB, aceI,* and *amvA* EPS genes in ST25 *A. baumannii* after 0, 10, or 15 min of serum exposure. The significance of the differences was calculated using two-way ANOVA (**P-values < 0.01, ***P-values < 0.001).

Considering that ATCC 19606 isogenic deletion mutants of *adeB, adeJ, aceI*, and *amvA* EPs genes displayed significantly reduced serum resistance compared to the wild-type strain (Fig. S5), we investigated whether EPs played a role in serum resistance of ST25 *A. baumannii* isolates and whether expression of EP genes was modulated during serum exposure. In CIVd isolates, transcripts of *adeB*, *adeJ,* and *aceI* genes were 80-fold higher (*P* < 0.001) compared to ATCC 19606 (Fig. 8). Furthermore, the *amvA* gene was upregulated up to 15-fold compared with ATCC 19606. Similarly, Clade III isolate 741019 exhibited 4- and 10-fold upregulation of *aceI* and *adeB* genes, respectively. In contrast, the expression of *aceI* and *adeB* genes in CIVc isolates was downregulated by 2- and 6-folds, respectively, compared with ATCC19606 *A. baumannii* (Fig. 8). No statistical correlation was found between the expression of *adeB*, *adeJ*, *aceI,* and *amvA* genes and resistance to serum exposure in ST25 *A. baumannii* isolates (Sperman *P*-values corresponding to 0.667, 0.677, 0.755, and 0.371, respectively).

## DISCUSSION

*A. baumannii* strains assigned to ST25 epidemic clonal lineage have been increasingly isolated worldwide from humans (5, 7, 15, 44), animals (5, 8), and the environment (6, 7) and were selected because of a high number of acquired and/or chromosomally embedded antimicrobial resistance genes (4–8, 15). In accordance with previous studies (5, 15), the core-genome MLST phylogeny of 203 ST25 isolates from human, animal, and environmental sources analyzed herein showed genomic diversity and distribution of genomes in four clades (CI–CIV) and four sub-clades (CIVa–CIVd). Corroborating previous publication by Lupo et al. on 141 ST25 *A. baumannii* isolates (5), the data of the present study showed geographical clustering, with CI and CIII clades over-representing isolates from South America, while isolates from CII and CIV clades came from all over the world. Additional epidemiological information was provided by KL and OCL. The capsular polysaccharides KL14, KL139, KL37, and KL116, which are a group of related structures found in ST25 *A. baumannii* isolates (45, 46), were identified in 47%, 10%, 7.4%, and 2% of ST25 *A. baumannii* isolates, respectively. The oligosaccharides OCL6, OCL5, and OCL10 were found in 50%, 35%, and 9.8% of ST25 *A. baumannii* isolates, respectively. In accordance with previous studies (18, 19), ST25 *A. baumannii* isolates included in this study showed an elevated variability of KL and OCL types. Nevertheless, KL14/OCL6 and KL14/OCL5 were the most prevalent KL and OCL types and were found in 61 and 11 out of 203 genomes, respectively. In keeping with our data, KL116/OCL6 and KL14/OCL6 genotypes are found in ST25 *A. baumannii* clinical isolates from Nigerian hospitals (16) and ST25 *A. baumannii* isolates from French companion animals (10), respectively.

Increasing evidence indicates that *A. baumannii* strains belonging to the ICLs I–III and to additional epidemic clonal lineages, including the ST25 lineage, possess peculiar virulence features, which sustain infection of animated hosts and survival in the environment (3, 20). The analysis of virulence genes identified in the core and shell genome of *A. baumannii* shows that all 203 ST25 *A. baumannii* genomes possess an extended panel of virulence genes belonging to biofilm, adherence, EPs, quorum sensing, metabolism/nutrition, immune modulation, and exotoxin categories. The analysis of the accessory genome of ST25 *A. baumannii* isolates showed the presence of tartrate metabolism genes only in CII genomes and gene families associated with phage-related functions in CIV genomes, which are absent or rare in other clades. Clade IV semi-specific genes were mostly phage derived and may influence surface structures, prophage dynamics, and host interactions, supporting the hypothesis that accessory elements contribute to the evolutionary diversification of this clade. Interestingly, ST25 *A. baumannii* genomes constituting the CIV clade were also the most recent and carried multiple resistance genes, including carbapenemase-encoding genes (5).

Further information on the virulence features of ST25 *A. baumannii* isolates was obtained by the phenotypic virulence-related and stress-related traits of a representative data set of 40 ST25 *A. baumannii* strains selected among the most prevalent genotypic profiles of ST25 genomes (Table S4). The analysis of virulence profiles in the *G. mellonella* model demonstrated that ST25 *A. baumannii* strains possess an intermediate/high infectivity, with CIVb and CVd strains being more virulent than strains assigned to other clades. The ability of ST25 CIVb and CIVd strains to kill *G. mellonella* larvae is similar to that of AYE *A. baumannii* strain assigned to ICL-I and to ATCC19606 (ST52) A. *baumannii* reference strains, while it is lower than that of ACICU strain *A. baumannii* assigned to ICL-II. Moreover, ST25 *A. baumannii* strains with KL14/OCL6 genotype showed higher ability to infect *G. mellonella* larvae than strains with other capsular types.

Virulence-related traits such as biofilm formation on abiotic surfaces, tolerance to oxidative stress, and resistance to desiccation contribute to the survival and spread of *A. baumannii* in the contaminated environment (3). In accordance with previous data (15, 43), ST25 *A. baumannii* isolates were intermediate biofilm producers but showed higher biofilm formation than the AYE strain and the ATCC17978 biofilm non-producer reference strain. A high biofilm production was observed in ST25 *A. baumannii* isolates with KL14/OCL5 and KL139/OCL6 genotypes, suggesting that the above KL/OCL loci may play a role in biofilm formation.

Our study also showed that ST25 *A. baumannii* strains, similarly to *A. baumannii* strains AYE (ICL-I), ACICU (ICL-II), and ATCC 19606 reference strains, possessed an elevated ability to survive desiccation on abiotic surfaces. While strains assigned to clades CI and CII exhibited variability in desiccation survival from 30 to 50 days, the single CIII isolate, and all isolates assigned to clades CIVb, CIVc, and CIVd showed desiccation survival up to 50–60 days. This enhanced desiccation resistance suggests that isolates assigned to clades CIVb-c-d possess adaptive mechanisms that promote long-term persistence on dry surfaces. The above findings are in agreement with previous data showing that ST25 *A. baumannii* strains possess elevated but variable resistance to desiccation (20), showing a similar phenotype to that found for AYE and ACICU strains (20, 47). Our data also demonstrate that ST25 *A. baumannii* isolates with KL14 and KL139 capsular polysaccharides have elevated resistance to desiccation compared to those displaying KL37, KL9, and KL130. This suggests that distinct capsule structures play a role in resistance to desiccation, as indicated by previous publications (48, 49).

In agreement with a previous study (38), *A. baumannii* ST25 strains possessed the ability to resist oxidative stress. The data of the present study demonstrated that tolerance to H_2_O_2_ differed according to the genotype of strains, CIVb and CIVd strains showing higher levels of resistance than those assigned to other clades and subclades. Furthermore, ST25 strains with KL14 and KL9 capsular loci possessed higher resistance to oxidative stress compared to those presenting the KL139, KL37, and KL130 capsular loci. ST25 *A. baumannii* isolates assigned to different clades were similarly resistant to serum exposure, except for isolates assigned to clade CIVc, which displayed significantly reduced serum resistance. On the other hand, ST25 A. *baumannii* strains with KL14/OCL6 and KL139/OCL6 genotype showed higher resistance to serum exposure than strains with KL14/OCL5 and KL9/OCL10 capsular type.

Overall, our data demonstrated that phenotypic virulence-related and stress-related traits of ST25 *A. baumannii* isolates depend on their KL and OCL genotypic profiles. In detail, the KL14/OCL6 profile emerged as a dominant, highly virulent, and multi-stress resistant genotype, KL14/OCL5 and KL9/OCL10 were specialized in biofilm formation, and KL139/OCL6 combined biofilm formation with environmental resilience (Table S4).

Previous studies demonstrated that the activation of EPs belonging to PACE, RND, and MFS efflux systems in *A. baumannii* modulates susceptibility to antimicrobials and biocides (50–52) as well as stress response, biofilm formation, and virulence (50–52). In support of this, the data shown herein demonstrated that *ΔadeB* and *ΔadeJ* (RND), *Δamva* (MFS), and *Δacel* (PACE) ATCC 19606 mutants possess reduced long-term survival to desiccation compared with the ATCC19606 wild-type strain, the maximum effect being observed in the *ΔadeB* mutant. Similarly, *∆adeB*, *∆adeJ, ∆aceI,* and *∆amvA* ATCC 19606 mutants showed reduced tolerance to H₂O₂ treatment, with *∆adeJ* and *∆adeB* strains being highly susceptible to oxidative stress. The data presented herein are consistent with the study of Srinivasan et al. (53), which demonstrates that inactivation of AbaO TolC-like protein (AbaO), an outer membrane protein often associated with RND-type efflux families in *Acinetobacter spp*. (50), increases sensitivity to oxidative stress challenge in *A. baumannii* strain AYE (53). The role of EPs in response to H₂O₂ treatment of ST25 *A. baumannii* strains was corroborated by the finding that gene expression of all EPs is upregulated in CIVb and CIVd isolates, which were ST25 *A. baumannii* strains highly tolerant to oxidative stress. Furthermore, a statistically significant correlation was found between *adeB* and *adeJ* expression and tolerance to oxidative stress in ST25 *A. baumannii* strains. We speculate that these efflux systems contribute directly or indirectly to oxidative stress protection, potentially by exporting toxic compounds or maintaining redox balance.

### Conclusions

*A. baumannii* epidemic clonal lineages are selected because of multidrug resistance and virulence and stress tolerance mechanisms, which contribute to infection in human and animal hosts and environmental persistence (3, 20). *A. baumannii* strains belonging to ST25 epidemic clonal lineage possess similar virulence profiles in *G. mellonella* infection model compared with *A. baumannii* strain AYE assigned to international clone I, but lower virulence profiles compared with *A. baumannii* strain ACICU assigned to international clone II. On the other hand, ST25 *A. baumannii* shows high resistance to desiccation, oxidative stress, and serum treatment, similarly to *A. baumannii* AYE and ACICU strains assigned to international clones I and II, respectively. ST25 *A. baumannii* strains showed an elevated heterogeneity in phenotypic profiles of virulence and stress resistance among CI–CIV and within the same clade. Also, virulence-related and stress-related traits of ST25 *A. baumannii* correlated with KL14, KL9, and KL139 types, OCL5, OCL6, and OCL10 types. Furthermore, the increased expression of AdeB and AdeJ RND EPs correlated with resistance to oxidative stress and serum in KL14/OCL6 ST25 CIVb and KL14/OCL6 ST25 CIVd *A. baumannii* isolates, suggesting that EPs regulate tolerance to stresses and survival in the contaminated environment.

## Data Availability

The original contributions presented in the study are included in the article/Supplementary material; further inquiries can be directed to the corresponding authors. The genomes analyzed in this study are available at the public project pubmlst.org/bigsdb?db=pubmlst_abaumannii_isolates&page=project&project_id=23.

## SUPPLEMENTAL MATERIAL

Supplemental figures msphere.00717-25-s0001.pdf Figures S1-S5.

Table S1 msphere.00717-25-s0002.xlsx ST25 *A. baumannii* genome database and KL/OCL analyses.

Table S2 msphere.00717-25-s0003.xlsx List of 127 virulence genes identified in 203 *A. baumannii* ST25 genomes.

Table S3 msphere.00717-25-s0004.xlsx Pangenome analysis matrix of ST25 *A. baumannii* genomes belonging to CI-CIV.

Table S4 msphere.00717-25-s0005.xlsx Virulence-related traits of 12 representative ST25 *A. baumannii* genotypes.

ASM does not own the copyrights to Supplemental Material that may be linked to, or accessed through, an article. The authors have granted ASM a non-exclusive, world-wide license to publish the Supplemental Material files. Please contact the corresponding author directly for reuse.
