# Supplementary material for: Genomic and phenotypic analysis of ST25 A. baumannii identifies virulence-associated clades and capsular/outer core locus types
Source: mSphere. 2025 Dec 31;11(1):e00717-25. doi: 10.1128/msphere.00717-25 (PMC12838272; doi:10.1128/msphere.00717-25)

Supplementary Materials for Integrated genomic and phenotypic analysis of ST25 *Acinetobacter baumannii* isolates identifies clades and capsular polysaccharide and lipooligosaccharide outer core types associated with virulence features

Antonella Migliaccio[1,2], Thibault Destanque[2], Marisa Haenni[2], Jean-Yves Madec[2], Keith A Jolley,[3] Maria Stabile[1,4], Eliana De Gregorio[4], Agnese Lupo[2] and Raffaele Zarrilli[1]

[1]Department of Public Health, University of Naples Federico II, Naples, Italy;

[2]ANSES - University of Lyon, Unité Antibiorésistance et Virulence Bactériennes, Lyon, France; [3]Department of Biology, University of Oxford, Oxford, United Kingdom;

[4]Department of Molecular Medicine and Medical Biotechnology, University of Naples Federico II, Naples, Italy.

This file includes the following:

Supplementary Figures

Figure S1. Pangenome structure and clade-specific shell genes in ST25 *A. baumannii* genomes.

Figure S2. Kaplan–Meier survival curves of *G. mellonella* larvae infected with 1 (±0.15) × 10^7 CFU/mL of ATCC 19606 and EPS mutants.

Figure S3. Desiccation resistance of ATCC 19606 and EPS mutants.

Figure S4. Oxidative stress tolerance of ATCC 19606 and EPS mutants.

Figure S5. Serum resistance of ATCC 19606 and EPS mutants.

Figure S1. Pangenome structure and clade-specific shell genes in ST25 *A. baumannii* genomes.

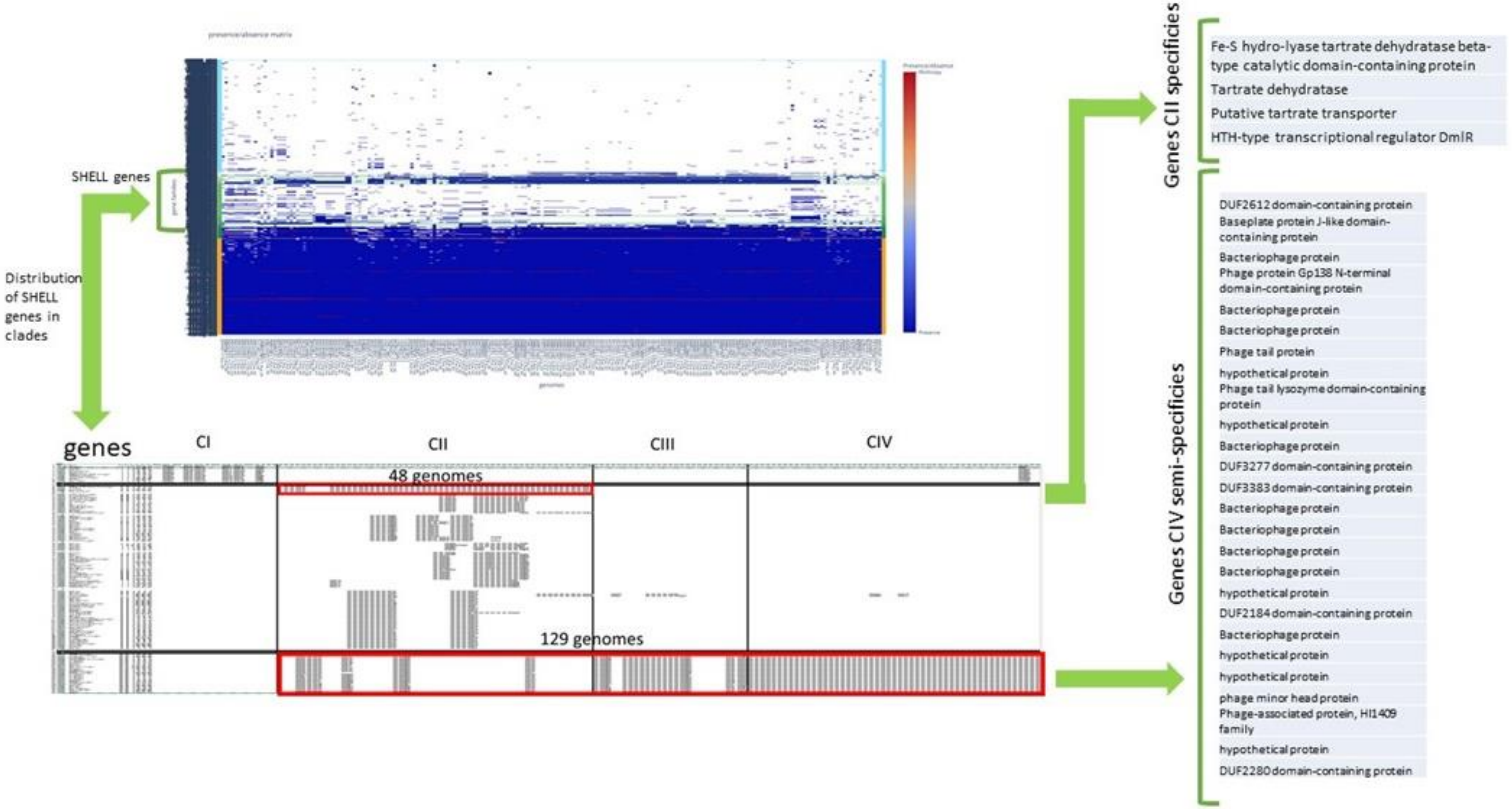

Presence/absence matrix of gene families across 203 *A. baumannii* ST25 genomes, categorized into persistent (yellow), shell (green), and cloud (blue) partition. Each column represents a genome, and each row a gene family; blue shading indicates gene presence. Selection of shell genes identified as specific or semi-specific to clades CII and CIV, respectively.

Figure S2. Kaplan–Meier survival curves of *G. mellonella* larvae infected with 1 (±0.15) × 10^7 CFU/mL of ATCC 19606 and EPS mutants.

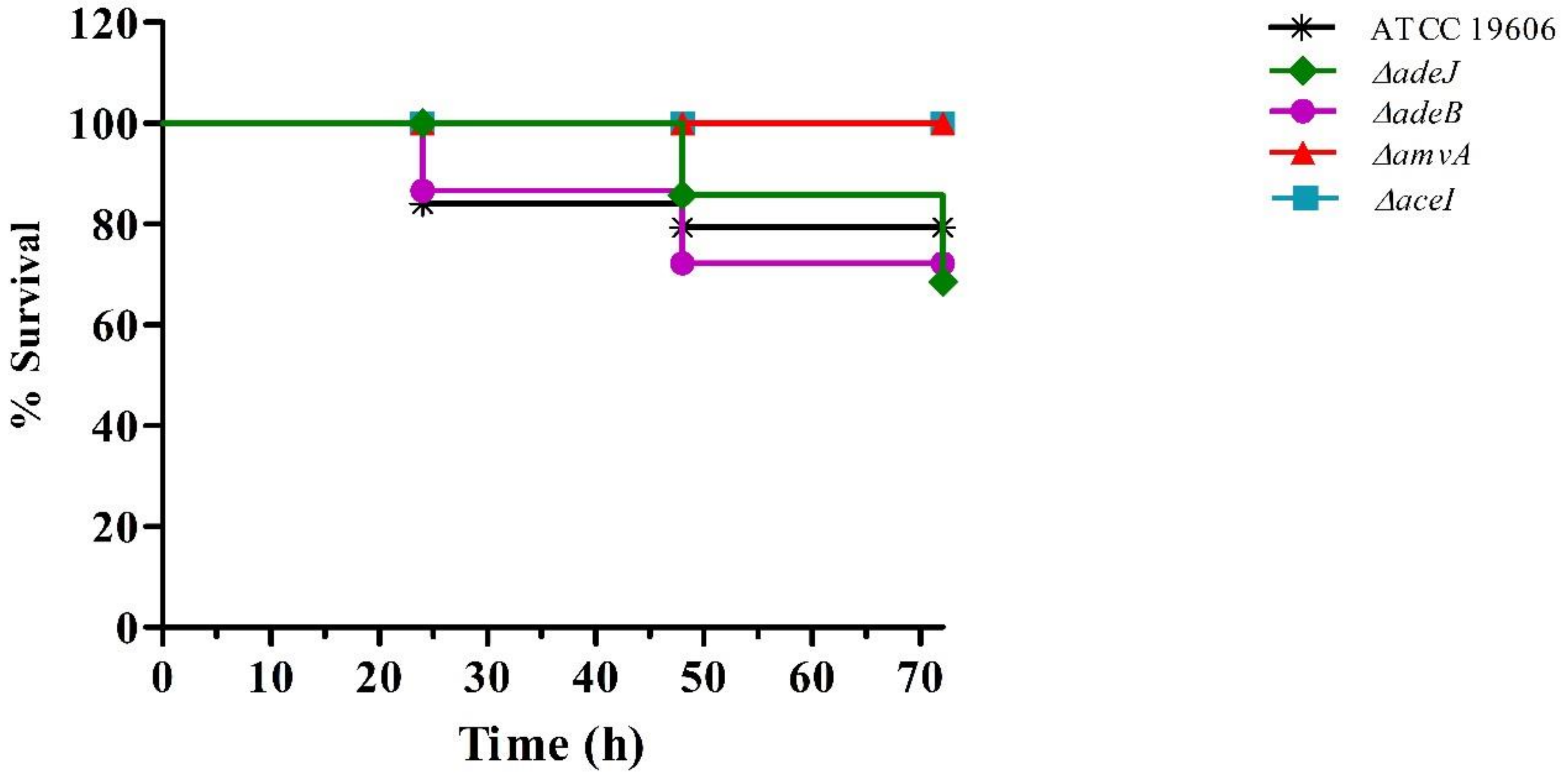

Figure S3. Desiccation resistance of ATCC 19606 and EPS mutants.

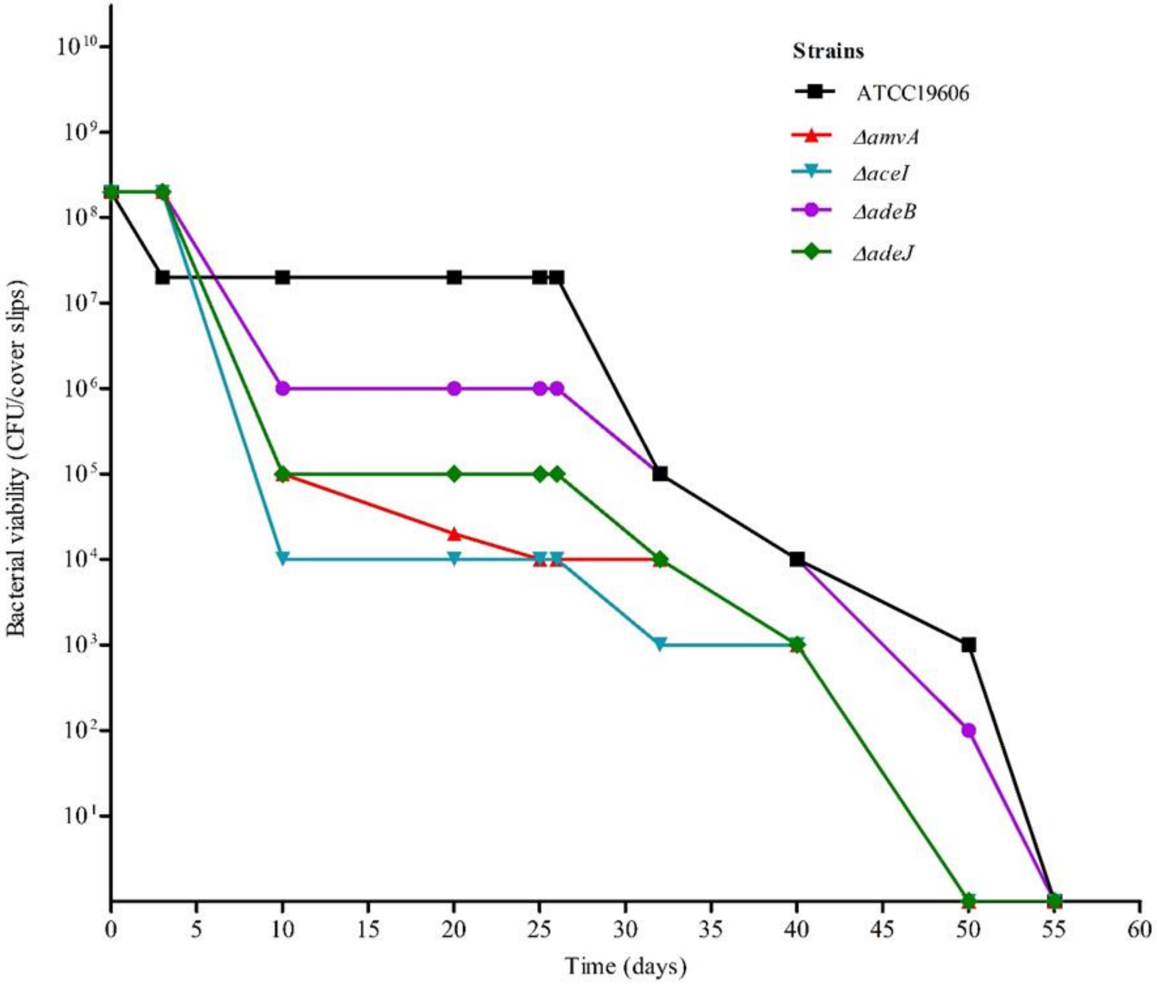

Figure S4. Oxidative stress tolerance of ATCC 19606 and EPS mutants.

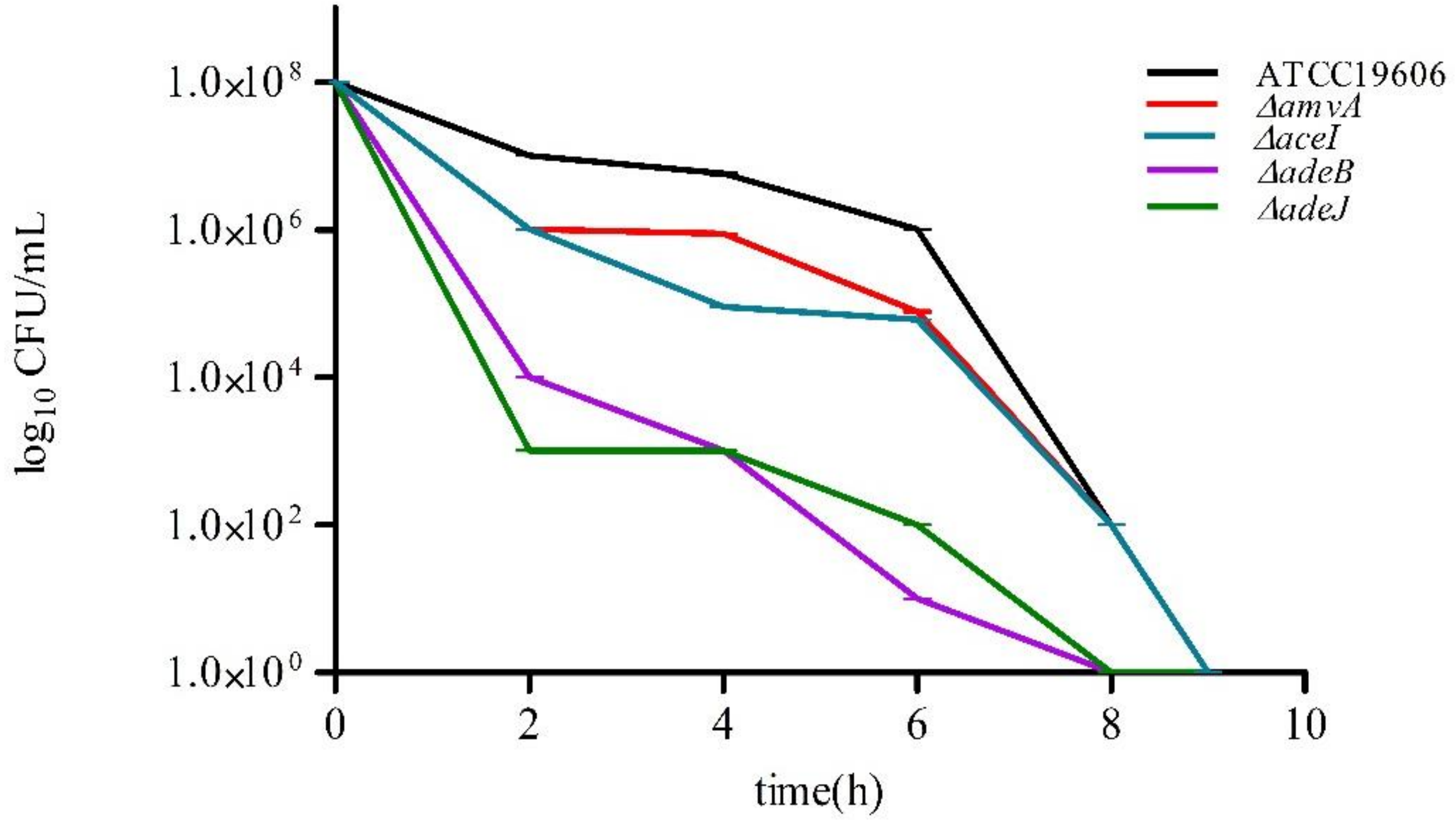

Figure S5. Serum resistance of ATCC 19606 and EPS mutants.

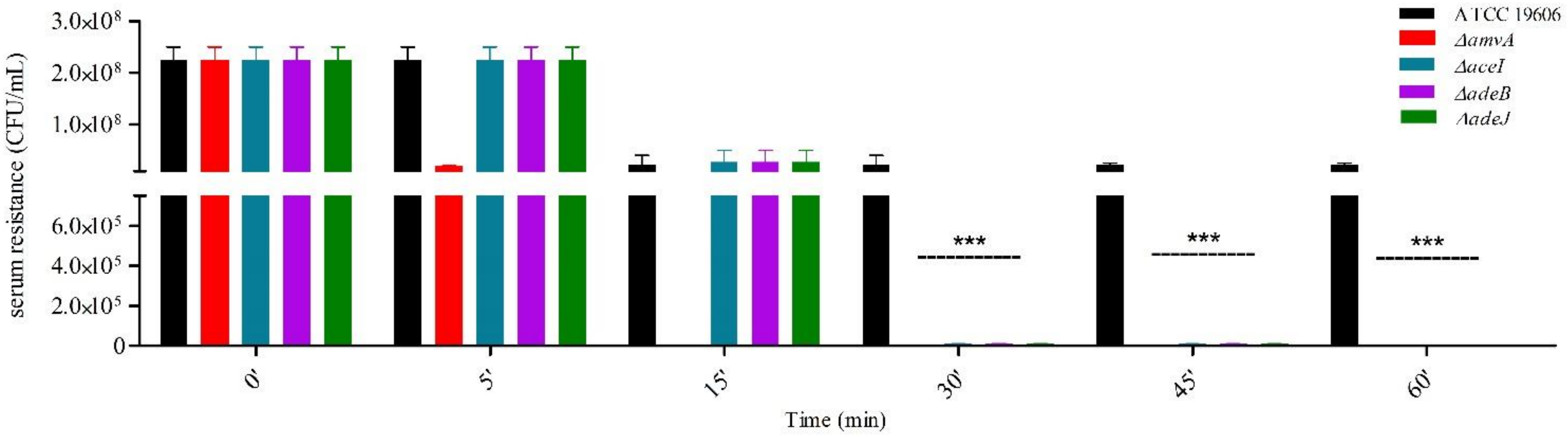

Supplement: Supplemental figures — Figures S1-S5. [file msphere.00717-25-s0001.pdf]
